# Meningitis in a Chinese adult patient caused by *Mycoplasma hominis*: a rare infection and literature review

**DOI:** 10.1186/s12879-016-1885-4

**Published:** 2016-10-12

**Authors:** Menglan Zhou, Peng Wang, Sharon Chen, Bin Du, Jinlong Du, Fengdan Wang, Meng Xiao, Fanrong Kong, Yingchun Xu

**Affiliations:** 1 Department of Clinical Laboratory, Peking Union Medical College Hospital, Chinese Academy of Medical Sciences, Beijing, 100730 People’s Republic of China; 2 Graduate School, Peking Union Medical College, Chinese Academy of Medical Sciences, Beijing, China; 3 Centre for Infectious Diseases and Microbiology Laboratory Services, Pathology West, Westmead Hospital, University of Sydney Darcy Road, Westmead, New South Wales, 2145 Australia; 4 Department of Medical Intensive Care Unit, Peking Union Medical College Hospital, Chinese Academy of Medical Sciences, Beijing, China; 5 Department of Clinical Laboratory, Fu Xing Hospital, Capital Medical University, Beijing, China; 6 Department of Radiology, Peking Union Medical College Hospital, Chinese Academy of Medical Sciences, Beijing, China

**Keywords:** *Mycoplasma hominis*, Post-operative infection, Meningitis, Hospital acquired pneumonia, Case report

## Abstract

**Background:**

*Mycoplasma hominis,* a well known cause of neonatal infection, has been reported as a pathogen in urogenital infections in adults; however, central nervous system (CNS) infections are rare. We report here the first case of *M. hominis* meningitis in China, post neurosurgical treatment for an intracerebral haemorrhage in a 71-year-old male.

**Case presentation:**

We describe a 71-year-old man who developed *M. hominis* meningitis after neurosurgical treatment and was successfully treated with combined azithromycin and minocycline therapy of 2 weeks duration, despite delayed treatment because the Gram stain of cerebrospinal fluid (CSF) yielded no visible organisms. The diagnosis required 16S rDNA sequencing analysis of the cultured isolate from CSF. Literature review of *M. hominis* CNS infections yielded 19 cases (13 instances of brain abscess, 3 of meningitis, 1 spinal cord abscess and 1 subdural empyema each). Delay in diagnosis and initial treatment failure was evident in all cases. With appropriate microbiological testing, antibiotic therapy (ranging from 5 days to 12 weeks) and often, multiple surgical interventions, almost all the patients improved immediately.

**Conclusions:**

Both our patient findings and the literature review, highlighted the pathogenic potential of *M. hominis* together with the challenges prompted by rare infectious diseases in particular for developing countries laboratories with limited diagnostic resources.

**Electronic supplementary material:**

The online version of this article (doi:10.1186/s12879-016-1885-4) contains supplementary material, which is available to authorized users.

## Background

*Mycoplasma hominis*, initially described as pleuropneumonia-like organism, is a commensal of the human oral cavity, respiratory tract, and genitourinary tract [1–3]. However, its role in the pathogenesis of infections in adult patients, especially extragenital infections such as central nervous system (CNS) infection, post-operative wound infections, mediastinitis, and septic arthritis [2, 4–7], has been difficult to determine. *M. hominis*, which does not possess a cell wall and hence is not identifiable by Gram staining of clinical specimens, is difficult to detect [3, 8, 9]. Culturing *M. hominis* which is fastidious in nature, is both resource- and time-consuming because specialized media and incubation conditions are required. Direct 16S rDNA PCR amplification/sequencing on clinical specimens may be performed but sensitivity is moderate at best, and not all laboratories perform this test [2, 3, 8]. The true incidence of *M. hominis* infections is thus probably underestimated and delayed diagnosis leads to delayed treatment with suboptimal outcomes [10].

CNS infections due to *M. hominis* are rare in patients other than neonates. To the best of our knowledge, only 19 cases of such infections have been reported in the English literature (Table 1). We herein report a case of *M. hominis* meningitis in which the organism was detected in the cerebrospinal fluid (CSF) following neurosurgical intervention for cerebral haemorrhage. This is the first reported case of meningitis in an adult caused by *M. hominis* from China.

**Table 1:** Literature reports of CNS infections caused by *Mycoplasma hominis* in non-neonatal patients (1950-2016.7)

No.	Author & year	Pt age(years), sex	Pt Country	HU	Tr	SI	Clinical manifestation	Days to Dx after Ad	Dx basis	Antibiotics used prior to diagnosis	Final antibiotic regimen	Otc	Ref
1	Paine et al. [14]	20, M	USA	N	Y	Y	fever, headache, a stiff neck	18	C	S + P + St	Sm	CR	[14]
2	Payan et al. [15]	29, M	USA	Y	Y	Y	fever, loss of consciousness	23	C	O + Cp + N + C	T + E	CR	[15]
3	Madoff S et al. [16]	11, F	USA	N	N	Y	fever	26	GIT + IRT	V + E	Mc	De	[16]
4	McMahon et al. [24]	76, M	USA	N	N	Y	fever, unresponsive	18	C	P + G	none	De	[24]
5	Kersten RC et al. [17]	20, M	USA	Y	Y	Y	fever, comatose	19	C	A + M + Su + V + Cf	M + Cf + Az + Am + Cl	Re	[17]
							NM	35	C	Az + Am + Cl	M + Cf + D + Cd	CR	[17]
6	Cohen & Kubak. [25]	18, F	USA	N	Y	Y	fever, altered mental status	20	C	E	D + Ci + C	CR	[25]
7	Zheng et al. [18]	22, F	USA	N	N	Y	fever, left-sided weakness and numbness	18	IRT + IBA	Ct + N + Ca + M	none	CR	[19]
8	Douglas et al. [19]	17, F	Australia	N	Y	Y	fever, headache, photophobia, nausea, vomiting	13	C + 16S	A + Ca + E	D + Cd	CR	[19]
9	House P et al. [20]	40, F	Spain	N	N	Y	headache, left facial weakness, nausea, afebrile	12 + “several”	C + 16S	V + Cf + M	Ci + M	CR	[20]
10	Kupila L et al. [21]	40, M	Finland	N	Y	Y	haematuria and urine retention, confused	14	16S	none	T	CR	[21]
11	McCarthy & Looke. [3]	48, M	Australia	N	N	Y	fever	36	C + 16S	Cz + V	Ga + Cd	CR	[3]
12	McCarthy & Looke. [3]	17, F	Australia	N	Y	Y	fever	17	C	V + Me	Ga + Mo	CR	[3]
13	Al Masalma et al. [22]	41, F	Russia	N	N	Y	vertigo, coma headache, hemiparesis	10	16S	V + Me	D	CR	[22]
14	Lee et al. [8]	48, F	Netherlands	Y	Y	Y	fever	15	16S	F + V	Mo	CR	[8]
15	Henao-Martínez et al. [10]	40, M	Somalia	N	Y	Y	fever	17	C + 16S	V + PT + Ct + M	D	CR	[10]
16	Pailhorie ‘s et al. [23]	43, M	France	N	Y	Y	fever, delirium tremens	13	Vitek MS + 16S	Me + V + Fo	L + D	CR	[23]
17	Whitson WJ [2]	17, M	USA	Y	Y	Y	fever, bicep and deltoid weakness	32	C	PT + V + Ct + M	D + Mo	CR	[2]
18	Hos NJ [27]	21, F	Germany	N	N	Y	fever, neck pain, nausea, vomiting,	31	C + 16S	A + Ct	Mo	CR	[27]
19	Reissier S [26]	39, M	France	N	Y	Y	afbrile, loss of consciousness	33	C + 16S + RT-PCR	PT + Li + Ct + Me + V	Mo	De	[26]
20	Present study	79, M	China	N	N	Y	fever, anepia and right-sided weakness	17	16S	Me + V + CF	Az + D + Mi	CR	

*Ad* admitted, *C* culture, *CR* clinical recovery, *De* death, *Dx* diagnosis, *GIT* growth inhibition test, *HU* hormone use, *IBA* immunoblot assay, *IRT* immunofluo-rescence test, *Otc* outcome, *Re* recurrence, *Ref* reference, *SI* surgical intervention, *16S* 16S rDNA sequencing, *RT-PCR* real-time PCR, *Tr* trauma, yrs: years

Ampicillin, (*A*) amoxicillin, (*Am*) azithromycin, (*Az*) chloramphenicol, (*C*) cefazolin, (*Ca*) clindamycin, (*Cd*) cefotaxime, (*Cf*) ciprofloxacin, (*Ci*) clavulanate potassium, (*Cl*) cephalothin (*Cp*), ceftriaxone (*Ct*), ceftazidime (*Cz*), cefoperazone/ sulbactam (*CF*), doxycycline (*D*), erythromycin (*E*),flucloxacillin (*F*), fosfomycin (*Fo*),gentamicin (*G*), gatifloxacin (*Ga*),levofloxacin (*L*), linezolid (*Li*), metronidazole (*M*), methacycline (*Mc*), meropenem (*Me*), minocyline (*Mi*), moxifloxacin (*Mo*),nafcillin (*N*), oxacillin (*O*),penicillin (*P*), piperacillin/tazobactam (*PT*), sulfadiazine (*S*), streptomycin (*Sm*), sulfathiazole (*St*), sulbactam (*Su*), tetracycline (*T*), vancomycin (*V*)

## Case presentation

### History and first admission

The patient was a 71-year-old man with a history of hypertension for 2 years who suddenly developed aphasia, and right-sided weakness and numbness while lifting water and was sent to the local hospital immediately on 21 September 2014. A cerebral computed tomography (CT) scan identified a cerebral hemorrhage rupturing into the ventricular system. He underwent craniotomy and evacuation of the hematoma. One week after the surgery, the patient developed hospital-acquired pneumonia, which was complicated by respiratory failure despite treatment with broad-spectrum antibiotics of imipenem.

### MICU admission

The patient was soon transferred to the medical intensive care unit (MICU) of Peking Union Medical College Hospital (PUMCH) on October 14. On admission, he was febrile, comatose (Glasgow Coma Score of 9), dyspneic, and hypotensive. Mechanical ventilation was started after endotracheal intubation.

### Examination on MICU & anti-infectious therapy

**Fig. 1:**
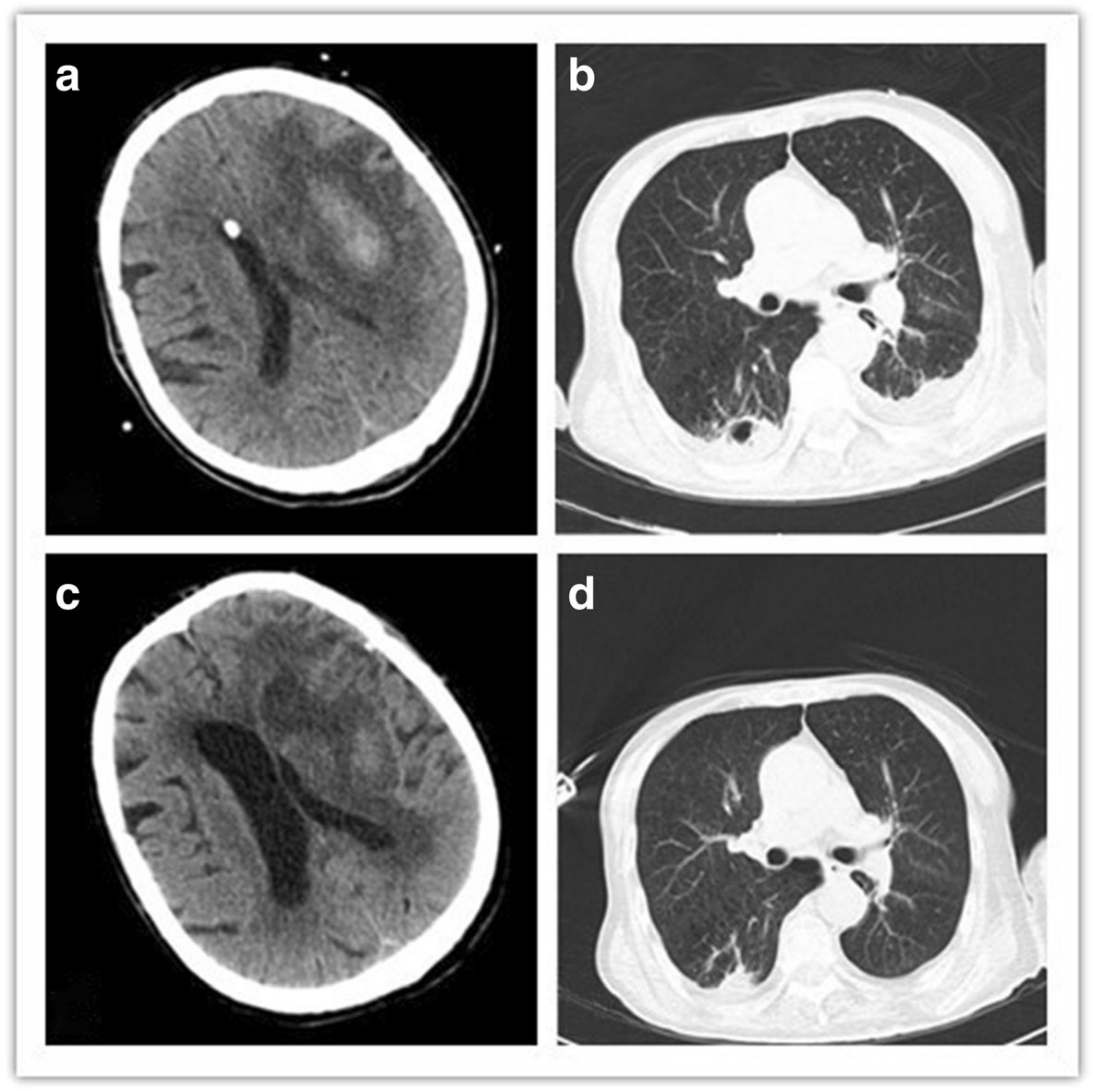
Computed tomography (CT) scans of the patient’s brain and lung during hospitalization. **a**. Cerebral CT scan revealed an extensive left hematoma in the temporal region of the hemispheres and moderate lateral ventricle enlargement with a drainage tube in it. The hematoma was surrounded by brain edema, narrowed gyri and the right–shifted cranial midline. **b**. Thoracic CT scan (lung windows) revealed a cavity with a wall of 7-mm thick surrounded by patchy shadowing in the right lower lobe. **c**. After 30 days of therapy, resolving brain swelling was seen in the CT scan with decreased edema. **d**. After 30 days of therapy, thoracic CT scan (lung window) revealed that the area of cavitation had decreased substantially

Empiric antibiotic treatment consisting of meropenem (2.0 g intravenously q8h) and vancomycin (1.0 g intravenously q12h) was initiated for hospital-acquired pneumonia. A lumbar puncture showed an opening pressure > 33 cmH_2_O, 1201 x 10^6^/L red cells, 201 x 10^6^/L white cells (79 % polymorphonuclear forms), an undetectable glucose concentration, protein 2.32 g/L, and chloride 128 mmol/L. No organisms were seen on Gram stain of the CSF. A cerebral CT showed an extensive left temporal hematoma and moderate lateral ventricle enlargement (Fig. 1a).

**Fig. 2:**
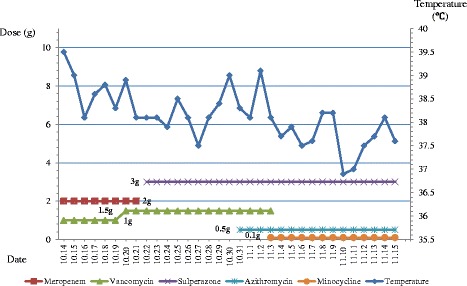
The correlation between the change of body temperature and the use of antibiotics during hospitalization

During his stay in ICU, repeated cultures of tracheal aspirates grew multidrug resistant (MDR) *Acinetobacter baumannii*, which was only susceptible to cefoperazone/sulbactam, and intermediately susceptible to tigecycline and minocycline. A follow-up chest CT scan revealed a thick-walled cavity in the right lower lobe (Fig. 1b). As a result, meropenem was changed to cefoperazone/sulbactam (3.0 g q8h), while the dose of vancomycin was increased to 1.5 g q12h to optimize the serum trough level (Fig. 2).

### Etiological examination

#### Microbiology laboratory examination

**Fig. 3:**
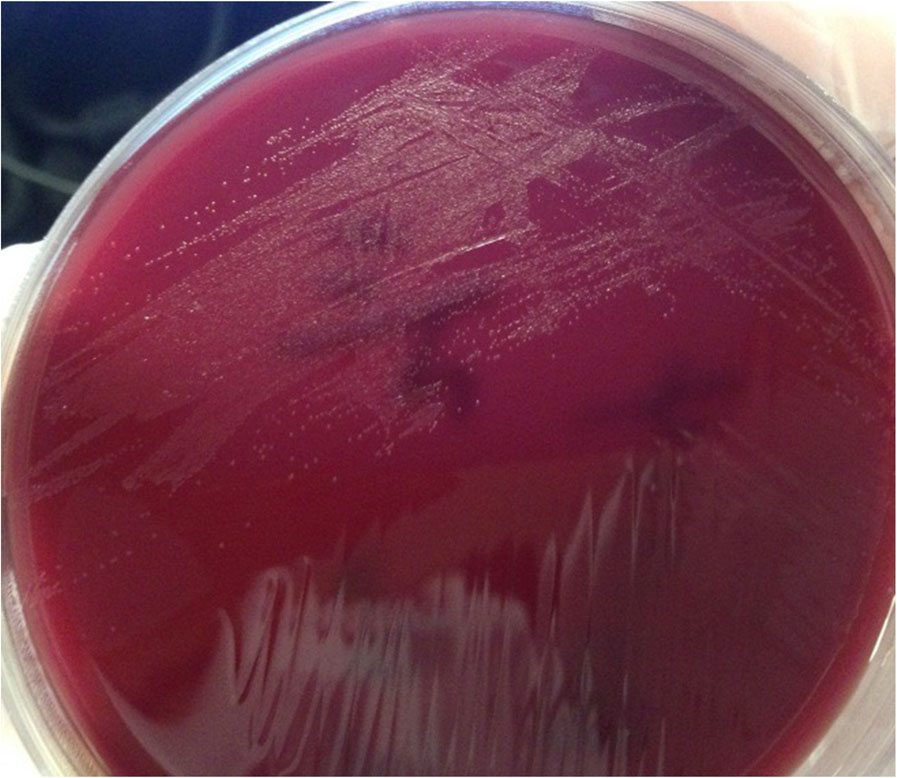
Non-hemolytic, semi-translucent pinpoint colonies of *M. hominis* were shown on 5 % blood sheep agar after 4 days of incubation

CSF specimens were plated onto 5 % sheep blood agar and chocolate agar and were incubated at 37 °C under aerobic and anaerobic conditions, and in air with 5 % CO_2_. On October 30, cultures revealed non-hemolytic, semi-translucent pinpoint colonies on sheep blood agar plate after 4 days of incubation under anaerobic conditions (Fig. 3). Gram-stain smears of the CSF sample showed no evidence of bacteria.

#### MALDI-TOF analysis of the cultured isolate

Two systems of the matrix-assisted laser desorption/ionization–time of flight mass spectrometry (MALDI-TOF MS) were used, i.e. the MALDI Biotyper (Bruker) and VITEK MS IVD (bioMérieux) as instructed by the manufacturer. The spectra were analyzed by using the MALDI Biotyper database library V.3.3.1.2 and the VITEK® MS IVD v2.0 database respectively. Both MALDI Biotyper and VITEK MS IVD consistently yielded no identifying profile and assigned “no identification” to this isolate.

#### 16S rDNA gene identification

Bacterial DNA was obtained from isolates by using QIAamp DNA minikit (Qiagen, Hilden, Germany) following the manufacturer’s instructions starting from 200 μL of bacterial pellet suspension about 2-3 McFarland followed by a series of extracting steps with different reagents. Amplification of the 16S rDNA gene was performed by broad-range bacterial polymerase chain reaction (PCR) assay using the universal primers: 27 F (5’-AGAGTTTGATCCTGGCTCAG-3’) and 1522R (5’-AAGGAGGTGATCCAGCCGCA-3’) as described before [11, 12]. Purified PCR products and sequencing primers (the same as for amplification) were mixed and sent to Ruibiotech (Beijing, China) for sequencing. Species identification was performed by comparing the obtained sequences against those in the GenBank database using the BLASTn software (http://www.ncbi.nlm.nih.gov/blast). By querying 16S rDNA sequences against those in the GenBank database, the isolate best matched with 3 *M. hominis* reference strains (GenBank accession numbers: CP011538.1, NR_041881.1 and CP009652.1) with an identity of 100 % (1385/1385), followed by *Mycoplasma equirhinis*, with an identity of 97 % (1314/1353). All *M. hominis* 16S rDNA nucleotide sequences available in GenBank till 2015 (*n* = 10) are summarized in Additional file 1.

### Post-treatment course

On November 2, the microbiology laboratory reported isolation from the CSF sample of *M. hominis*, which was susceptible to doxycycline, and intermediately suscepible to azithromycin by the bioMérieux® SA *Mycoplasma* IST2 kit (Biomerieux, France). Combination therapy with azithromycin (0.5 g qd) and minocycline (100 mg q12h) was then started (Fig. 2). On November 16, 14 days after appropriate antimicrobial therapy was started, the patient was transferred back to the local hospital. Repeated brain and chest CT scans before discharge showed marked improvement of the cerebral edema and size of the brain swelling (Fig. 1c), and almost complete resolution of the pulmonary cavity and pleural effusion (Fig. 1d).

## Discussion

A PubMed search was performed using the following key words: “*Mycoplasma hominis*” AND “encephalitis” OR “cerebritis” OR “cephalitis” OR “neuraxitis” OR “phrenitis” OR “meningitis” OR “brain abscess” OR “cranial infection” OR "myelitis" OR " polyradiculoneuritis " OR "spinal cord infection". A total of 58 manuscripts were found, most reporting *M. hominis* infection in neonates, which most likely arising from contact with maternal genital flora [13]. By carefully reading all the papers, we found that there were only 19 reported non-neonatal-associated cases of CNS infection caused by *M. hominis*; none was reported from China (Table 1). Of these 20 cases, brain abscess was the most common CNS infections (*n* = 13) [3, 10, 14–23], followed by meningitis (*n* = 4) [8, 24–26], spinal cord abscess (*n* = 1) [2], subdural empyema (*n* = 1) [27], (Table 1).

Current microbiological analysis, mostly based on direct examination and culture of pus specimens, underestimate the role of fastidious microorganisms, such as *M. hominis* [22], in CNS infections. Predisposing host factors such as immunosuppression, malignancy, trauma, and manipulation or surgery of the genitourinary tract are considered to be risk factors for extra-genital infections caused by this microorganism [8, 15, 17, 18, 28]. In most *M. hominis* brain infections thus far described, patients usually presented with prior head trauma or had undergone neurosurgical procedures [8, 10, 14, 15, 17, 19, 21, 23–27]. This was also the case in our patient who had an intracererbal haematoma evacuated 7 days prior to developing meningitis.

Three routes of intracranial infection are typically considered: direct contamination during trauma, direct contamination during surgery, or seeding of the cerebral site secondary to bacteraemia due to genitourinary manipulation [21, 23]. In traumatized brain tissue where CNS capillaries are damaged, *M. hominis* can easily reach the ischemic brain tissue through blood circulation. In our case, the patient was exposed to the aforementioned last 2 hypothetical sources. Cerebral hemorrhage was cured by a neurosurgical intervention, but he also underwent urinary catheterization after the coma during hospitalization. Therefore, we were unable to definitively identify the source of infection.

Identification of *M. hominis* infections by culture is challenging due to the slow growth of the colonies and the absence of cell wall, which contributes to a negative result on Gram staining [10], as in our patient. Moreover, even when culture is successful, it is difficult to rule out the possibility of contamination. Although *M. hominis*, being less fastidious than other mycoplasmas, is able to grow on conventional blood agar medium, specific laboratory methods are required for its identification. In most reports of cerebral infections, 16S rDNA sequencing is required for definitive identification as was the case here [8]. It has been recently highlighted that MALDI-TOF MS could be useful for the rapid identification of *M. hominis* [23, 29]. However, we were unable to identify *M. hominis* by MALDI TOF MS even though the spectra of this species is represented in the VITEK® MS IVD v2.0 database. In this regard, similar results were also found for the MALDI Biotyper by Nulens E and his colleges [30]. Thus, further improvement of *M. hominis* spectra database seems necessary. Nevertheless, neither the Bruker nor Vitek database misidentified the strain as another species.

Since cases of intracranial infections caused by *M. hominis* are rare, clinicians usually fail to consider the diagnosis in the absence of microbiological evidence. In addition, it is difficult to distinguish brain abscesses or meningitis caused by mycoplasmas from those caused by bacteria or viruses, due to the lack of specific clinical manifestations. This may lead to delayed initiation of antimicrobial therapy with serious clinical consequence [31], as *M. hominis* is not susceptible to most first-line antibiotics used to treat brain abscesses [21]. The possibility of a *M. hominis* infection should be suspected when Gram stain reveals abundant neutrophils but no bacteria, and empirical treatment shows poor efficacy during this period.

In all previously published case studies of *M. hominis* brain abscesses, treatment involved abscess drainage, debridement, and specific antimicrobial therapy. The importance of surgical treatment is evident from a case report of a patient who responded to surgical therapy alone [32]. Infections caused by *Mycoplasma* spp. (i.e. a microorganism lacking both cell wall and folic acid synthesis) require ad-hoc antibiotic treatment [33, 34]. There are no CLSI (Clinical and Laboratory Standards Institute) and EUCAST (European Committee on Antimicrobial Susceptibility Testing) breakpoints for *M. hominis* at present. Nevertheless, the organism is generally considered to be susceptible to tetracyclines, lincosamides, streptogramins and quinolones, but not to the macrolides although tetracyclines-resistant *M. hominis* has been reported [10, 35–37]. However, poor passage through the blood brain barrier could lead to low antibiotic concentrations in the brain, with the possible major exception of fourth generation quinolones that, besides exhibiting low MICs for *M. hominis*, do possess reasonable CSF penetration [35–43]. Because of the tendency for chronic and often latent infection, long-term antimicrobial treatment against *M. hominis* is warranted. However, surgical drainage and debridement remain the key to recovery, since patients may respond to surgical treatment alone [29].

In our case, initial treatment with meropenem and vancomycin showed little efficiency since a low-grade fever still persisted (as shown in Fig. 2). MDR *A. baumannii* was also isolated from the lower respiratory tract possibly due to nosocomial infection. Persistent fever despite various antibiotic treatment with meropenem, vancomycin, and cefoperazone/sulbactam, as well as the satisfactory response to azithromycin and minocycline, strongly suggested that *M. hominis*, rather than *A. baumannii* was the primary pathogen in our patient.

## Conclusions

The prevalence of brain infections caused by *M. hominis* may be increasing, presenting a diagnostic and therapeutic challenge to clinicians. We reported here the first case of meningitis caused by *M. hominis* in an adult in China, who was successfully treated with azithromycin and minocycline. The pathogenic potential of *M. hominis*, the need for early diagnosis, and the importance of initial appropriate chemotherapy must be highlighted also in developing countries, where the challenges in diagnostic capacity for clinical laboratories are greater.

## Additional file

Additional file 1: Near complete length GenBank *Mycoplasma hominis* 16S rRNA sequences analysis. A summary of all the *Mycoplasma hominis*16S rRNA sequences deposited in GeneBank. (DOCX 16 kb)

## Abbreviations

BBB: Blood brain barrier
CLSI: Clinical and laboratory standards institute
CNS: Central nervous system
CSF: Cerebrospinal fluid
CT: Computed tomography
EUCAST: European committee on antimicrobial susceptibility testing
MALDI-TOF MS: Matrix-assisted laser desorption/ionization–time of flight mass spectrometry
MDR: Multidrug resistant
MICU: Medical intensive care unit
PCR: Polymerase chain reaction
PUMCH: Peking union medical college hospital
