# Supplementary material for: Meningitis in a Chinese adult patient caused by Mycoplasma hominis: a rare infection and literature review
Source: BMC Infect Dis. 2016 Oct 12;16:557. doi: 10.1186/s12879-016-1885-4 (PMC5059901; doi:10.1186/s12879-016-1885-4)
Supplement: Additional file 1: — Near complete length GenBank Mycoplasma hominis 16S rRNA sequences analysis. A summary of all the Mycoplasma hominis16S rRNA sequences deposited in GeneBank. (DOCX 16 kb) [file 12879_2016_1885_MOESM1_ESM.docx]

**Additional file 1. Near complete length GenBank *Mycoplasma hominis*16S rRNA sequences analysis**

| No. | Strain | Country | Origin | 16S rRNA | | | References(PMID) |
| --- | --- | --- | --- | --- | --- | --- | --- |
|  |  |  |  | Genbank Accession No | Identity(%) | ST |  |
| 1 | PG21/ATCC 23114 | England | human genitalia | NR_041881.1 (=AF443616.3) =NR_074603.1 (=FP236530.1) | Type Strain | ST1 | 12234836 |
| 2 | ATCC 33131 | USA | human urethra | CP011538.1 | 1514/1514(100%) | ST1 | 26159538 |
| 3 | ATCC 27545 | USA | unknown | CP009652.1 | 1514/1514(100%) | ST1 | unpublished |
| 4 | NBRC 14850 | Japan | unknown | NR_113679.1  (=AB680681.1) | 1443/1443(100%) | ST1 | unpublished |
| 5 | 7488 | Denmark | human cervix | AJ002269.1 | 1514/1515(99%) | 161 C-T | 9734066 |
| 6 | P2 | Denmark | huaman urinary tract | AJ002268.1 | 1515/1515(100%) | ST1 | 9734066 |
| 7 | SC4 | England | huaman urethra | AJ002267.1 | 1515/1515(100%) | ST1 | 9734066 |
| 8 | DC63 | England | human cavum oris | AJ002266.1 | 1514/1515(99%) | 1202 C-T | 9734066 |
| 9 | 183 | USA | huaman vagina | M96660.1 | 1444/1457(99%) | 13-bp mismatch | 7684753 |
| 10 | AF1 | USA | human amniotic fluid | CP009677.1 | 1514/1515(99%) | 131 G-A | 25637842 |
| 11 | present article | China | human brain | NR_041881.1, CP011538.1,  CP009652.1 | 1385/1385(100%) | ST1 | present article |

16S rRNA Sequences (1385bp) in the present article:

TTTATAAGAGTTTGATCCTGGCTCAGGATGAACGCTGGCTGTGTGCCTAATACATGCATGTCGAGCGAGGTTAGCAATAACCTAGCGGCGAATGGGTGAGTAACACGTGCTTAATCTACCTTTTAGATTGGAATACCCATTGGAAACAATGGCTAATGCCGGATACGCATGGAACCGCATGGTTCCGTTGTGAAAGGCGCTGTAAGGCGCCACTAAAAGATGAGGGTGCGGAACATTAGTTAGTTGGTGAGGTAATGGCCCACCAAGACTATGATGTTTAGCCGGGTCGAGAGACTGAACGGCCACATTGGGACTGAGATACGGCCCAAACTCCTACGGGAGGCAGCAGTAGGGAATATTCCACAATGAGCGAAAGCTTGATGGAGCGACACAGCGTGCACGATGAAGGTCTTCGGATTGTAAAGTGCTGTTATAAGGGAAGAACATTTGCAATAGGAAATGATTGCAGACTGACGGTACCTTGTCAGAAAGCGATGGCTAACTATGTGCCAGCAGCCGCGGTAATACATAGGTCGCAAGCGTTATCCGGAATTATTGGGCGTAAAGCGTTCGTAGGCTGTTTGTTAAGTCTGGAGTTAAATCCCGGGGCTCAACCCCGGCTCGCTTTGGATACTAGCAAACTAGAGTTAGATAGAGGTAAGCGGAATTCCATGTGAAGCGGTGAAATGCGTAGATATATGGAAGAACACCAAAGGCGAAGGCAGCTTACTGGGTCTATACTGACGCTGAGGGACGAAAGCGTGGGGAGCAAACAGGATTAGATACCCTGGTAGTCCACGCCGTAAACGATGATCATTAGTCGGTGGAGAATCACTGACGCAGCTAACGCATTAAATGATCCGCCTGAGTAGTATGCTCGCAAGAGTGAAACTTAAAGGAATTGACGGGGACCCGCACAAGCGGTGGAGCATGTGGTTTAATTTGAAGATACACGGAAAACCTTACCCACTCTTGACATCCTTCGCAAAGCTATAGAGATATAGTGGAGGTTATCGGAGTGACAGATGGTGCATGGTTGTCGTCAGCTCGTGTCGTGAGATGTTTGGTCAAGTCCTGCAACGAGCGCAACCCCTATCTTTAGTTACTAACATTAAGTTGAGGACTCTAGAGATACTGCCTGGGTAACTGGGAGGAAGGTGGGGATGACGTCAAATCATCATGCCTCTTACGAGTGGGGCCACACACGTGCTACAATGGTCGGTACAAAGAGAAGCAATATGGCGACATGGAGCAAATCTCAAAAAGCCGATCTCAGTTCGGATTGGAGTCTGCAATTCGACTCCATGAAGTCGGAATCGCTAGTAATCGCAGATCAGCTATGCTGCGGTGAATACGTTCTCGGGTCTTGTACACACCGCCCGTCA
